# Atherosclerotic Renal Artery Stenosis: Should we Intervene Earlier?

**DOI:** 10.1007/s11906-018-0829-3

**Published:** 2018-04-10

**Authors:** Peter W. de Leeuw, Cor T. Postma, Wilko Spiering, Abraham A. Kroon

**Affiliations:** 1 Department of Medicine, Maastricht University Medical Center, Maastricht, The Netherlands; 2 Department of Medicine, Zuyderland Medical Center, Sittard-Geleen, Heerlen, The Netherlands; 3 Department of Medicine, University Medical Center Nijmegen St Radboud, Nijmegen, The Netherlands; 4 Department of Vascular Medicine, University Medical Center Utrecht, Utrecht University, Utrecht, The Netherlands

**Keywords:** Atherosclerosis, Renovascular hypertension, Cardiovascular complications, Renal function, Renal artery stenosis, Intervention

## Abstract

**Purpose of Review:**

Randomized trials have failed to show clinical benefit in patients with atherosclerotic renal artery stenosis who were treated with angioplasty with or without stenting. However, these studies were done in patients with a high-grade stenosis. This paper examines whether there are arguments to consider patients with low-grade stenosis for angioplasty.

**Recent Findings:**

Patients with low-grade (< 50%) atherosclerotic renal artery stenosis have an excess risk for cardiovascular and renal complications. This could be related to inflammatory factors being generated by the stenotic kidney. Moreover, even a kidney with low-grade stenosis clears less or produces more of the natural nitric oxide inhibitor ADMA.

**Summary:**

Patients with low-grade atherosclerotic renal artery stenosis have an increased risk for a variety of complications. In addition, the abnormality is progressive. There is a case for setting up a prospective trial to examine whether angioplasty confers benefit in patients with low-grade renal artery stenosis.

## Introduction

Atherosclerotic renal artery stenosis (ARAS) is a leading cause of secondary hypertension and has profound, deleterious effects on the cardiovascular system [1–3] as well as on the kidney [4•, 5]. Although there is general agreement that patients with ARAS should be vigorously treated with antihypertensive and lipid-lowering drugs, there is controversy regarding the role of angioplasty with or without stent placement. The outcomes of several randomized trials have challenged the view that angioplasty with or without stent placement is beneficial in these patients as such treatment was not superior to treatment with medication alone. Accordingly, there is a tendency now among clinicians not to look actively for renal artery stenosis as it is deemed to be without therapeutic consequences. However, negative trial results do not necessarily imply that the treatment under study should be abandoned altogether in all cases. Indeed, it is very well possible that the patients who were included in the trials had a specific phenotype that is not representative for all patients with the condition or that the results of angioplasty are more dependent upon the various stages of the disease. Detailed knowledge of the pathophysiology of renovascular abnormalities is necessary, therefore, to appreciate whether the available trial results can be applied to all patients with ARAS or only to a specific subset. Here, we will review briefly some of the recent findings regarding the natural history of ARAS and its pathophysiological sequelae. The central question which we will try to answer is whether our current understanding of the abnormality and its treatment provides enough guidance for the physician to base treatment decisions on in specific patient groups.

## Clinical Significance of Renal Artery Stenosis

While ARAS in all likelihood is part of a generalized atherosclerotic process, the excess cardiovascular morbidity and mortality cannot simply be explained on the basis of hypertension or degree of renal impairment. This can be substantiated by the following observations. Kalra and coworkers, for instance, have found that incident ARAS, i.e., an atherosclerotic stenosis that developed in a random population sample without prior renovascular disease, was associated with a significant increase in cardiovascular complications and mortality as compared to the general population [1]. There is also evidence that such cardiovascular abnormalities are more pronounced with bilateral than with unilateral ARAS [2, 6]. In addition, for the same level of blood pressure and degree of renal dysfunction, patients with ARAS have more cardiovascular comorbidity and a greater prevalence of left ventricular hypertrophy and diastolic dysfunction than those without ARAS [6]. Finally, the rate of complications increases already with low-grade stenosis, even as low as less than 30% luminal reduction [7••]. Generally speaking, such low-grade stenoses are considered to be of no hemodynamic significance but they may very well be clinically significant.

In an era where imaging possibilities are virtually endless, we are likely to find, every once in a while, a so-called abnormality which has no clinical significance whatsoever. One could argue that such may be the case when renal artery stenosis is found accidentally for example in normotensive patients who are scheduled for coronary angiography. Indeed, the presence of an ARAS does not always lead to hypertension as some early autopsy studies in unselected patients already showed [8, 9]. A more recent autopsy study reported a prevalence of 6.8% of unsuspected renal artery lesions in a normotensive general population older than 65 years without clinically evident renal disease [10]. Nevertheless, incidental ARAS appears to be associated with a significantly increased mortality and, therefore, can have major clinical significance [11]. It thus seems that ARAS is not simply an otherwise innocent aspect of a generalized atherosclerotic process, but rather a risk factor for cardiovascular complications in its own right. Once ARAS has developed, even if low-grade, this abnormality per se appears to act as an enhancer of atherosclerosis elsewhere in the body. It accelerates or aggravates vascular lesions in various organs, including the kidney, through mechanisms that at least partly appear to be independent from elevated blood pressure [12]. Also, renal function will be adversely influenced by the presence of ARAS, regardless of blood pressure and regardless of the degree of stenosis [13]. Therefore, even low-grade ARAS should potentially be considered as clinically significant.

## Pathophysiological Aspects

**Fig. 1:**
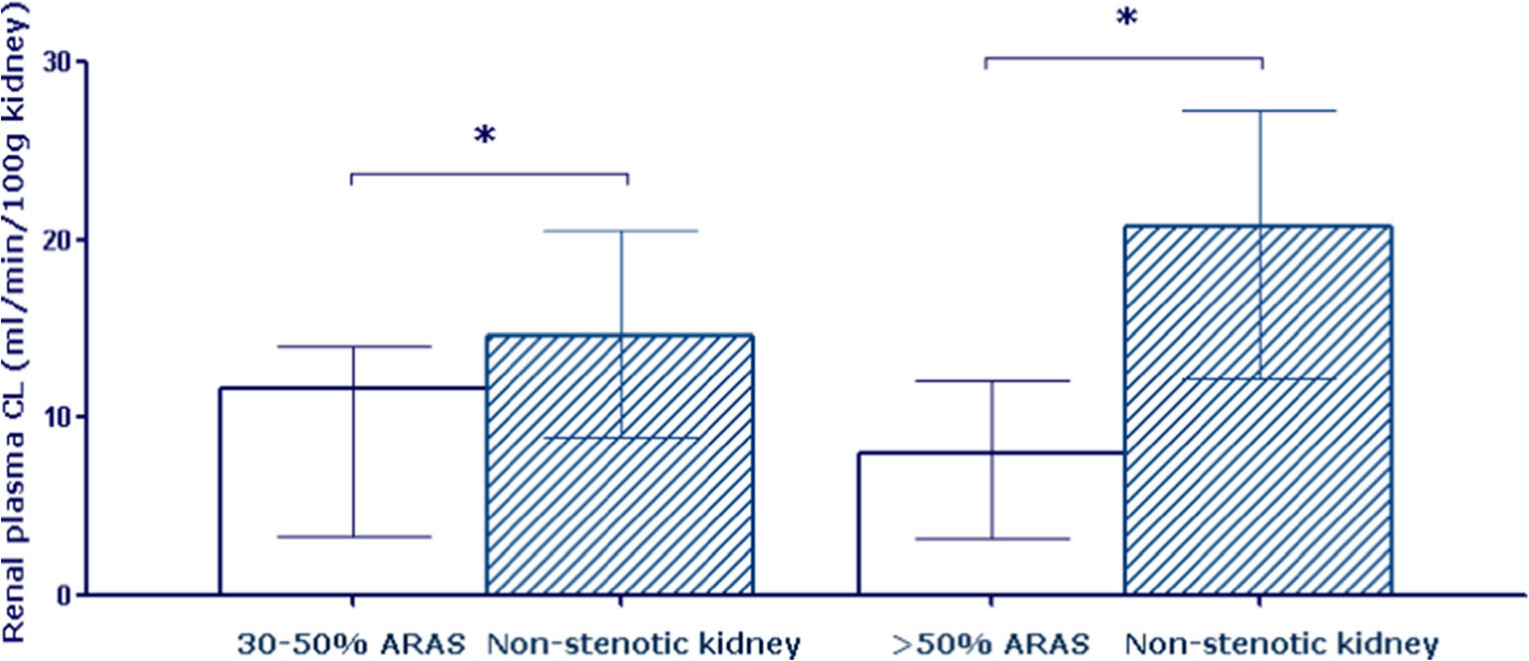
Reduced renal plasma clearance of ADMA by the stenotic kidney in patients with unilateral ARAS. Data from [17]

The excess amount of atherosclerosis in patients with ARAS suggests that the stenotic kidney entails pro-atherogenic processes with the renin-angiotensin system being an obvious mediator. However, in bilateral disease, renin levels are usually lower than in unilateral disease, yet the opposite is true for the incidence of cardiovascular complications [2, 6]. Sympathetic activation, locally triggered by the intrarenal production of hypoxia-related substances such as adenosine, could also play a role. Recent experimental studies also point towards hypoxia-induced pro-inflammatory, pro-oxidant, and pro-fibrinogenic mechanisms that are activated during repeated acute ischemic episodes and from which the kidney can recover as long as the stenosis is moderate [14••, 15••]. For instance, compared to normotensive subjects and patients with essential hypertension, those with unilateral ARAS and more than 60% stenosis have increased levels of the acute phase protein NGAL (neutrophil gelatinase-associated lipocalin) in their renal venous blood on both the stenotic and the contralateral side [16]. On the other hand, inflammatory markers such as tumor necrosis factor (TNF)-alpha and interferon-gamma are higher on the stenotic than on the contralateral side. These results have been interpreted as pointing to ongoing inflammatory processes or acute ischemic events within the post-ischemic kidney. However, whether such markers are also elevated in patients with lesser degrees of stenosis is uncertain. In our own laboratory, we have measured plasma levels of the endogenous nitric oxide inhibitor ADMA (asymmetric dimethylarginine) in systemic and renal venous blood of patients with varying degrees of unilateral ARAS [17]. Compared to patients with essential hypertension, carefully matched for age, sex, and blood pressure, systemic levels of ADMA were elevated in those with more than 50% stenosis only. However, the renal plasma clearance of ADMA was significantly lower in stenotic kidneys compared to the contralateral one in both high-grade and low-grade stenosis (Fig. 1). Moreover, plasma clearance of ADMA by the contralateral kidney is higher when the stenosis in the affected kidney is more severe. These data strongly suggest that a kidney with ARAS, even if the stenosis is low-grade, has a reduced capacity to clear the plasma of ADMA (or perhaps even produces this compound itself). The contralateral kidney tries to compensate for this pathophysiological abnormality by increasing its plasma clearance of ADMA and is able to keep systemic levels within relatively normal limits as long as the stenosis on the affected side is below 50%. As circulating ADMA is a pro-atherogenic substance, our results are compatible with the view that the kidney contributes to acceleration of the atherosclerotic process itself by inhibiting nitric oxide in the systemic vasculature. Although circulating levels of ADMA are not yet increased in patients with low-grade stenosis, it is obvious that even in these patients there are already significant pathophysiological changes occurring in the affected kidney. So far, however, we do not have sufficient information to know precisely what happens in humans during the phase of slowly-progressing luminal narrowing and whether targeting progressive stenosis therapeutically would confer benefit.

## Atherosclerotic Renal Artery Stenosis Is a Progressive Disorder

In an attempt to explore whether ARAS is a progressive disease, Schreiber and colleagues retrospectively reviewed data from 85 patients who had at least two angiographic evaluations over a 10-year period [18]. They found that in 44% of cases, the stenosis progressed with complete occlusion occurring in 16%. In a similar study by Tollefson and Ernst, progression of unsuspected atherosclerotic renal arterial stenoses occurred in 53% of the arteries [19]. Total occlusion developed in 9% of the arteries, all of which had a high-grade stenosis at baseline. The rate of diameter stenosis progression was approximately 5% per year, irrespective of the initial degree of stenosis. Although the diagnosis in both reports was based on angiographic films, which remain the gold standard, the studies had the disadvantage of being retrospective. Nevertheless, these data have subsequently been confirmed in prospective studies, albeit with non-invasive techniques. For instance, Zierler and associates followed 84 patients with at least one abnormal renal artery with repeat duplex scanning. The cumulative incidence of progression from less than 60 to 60% or greater stenosis was 23 and 42% respectively after 1 and 2 years [20]. In a later study from the same department, Caps and coworkers prospectively followed the fate of 295 kidneys in 170 patients who were referred for renal ultrasound because of hypertension, renal insufficiency or both, and found that the cumulative incidence of ARAS progression was 35% at 3 years and 51% at 5 years [21]. Risk of progression was greatest (49%) in those who at baseline had already more than 60% stenosis, while it was 28% in those with lesser degrees of stenosis. More importantly, 18% of those with initially normal renal arteries developed a stenosis. Factors that were significantly associated with the risk of progression were a systolic blood pressure above 160 mmHg, the presence of diabetes mellitus, and high-grade stenosis in either the ipsilateral or the contralateral kidney. Progression to total occlusion, however, was rare. It should be stressed, though, that all diagnoses were made by ultrasound which is not the most accurate technique to establish the degree of stenosis. In another analysis, the same investigators found that renal atrophy occurred in 21% of cases with high-grade stenosis, but also in nearly 6% of those with less than 60% stenosis [22]. Altogether, the available evidence suggests that in patients with ARAS, the stenosis progresses over time and that even patients with low-grade stenosis are at some risk of loss of parenchymal renal tissue. Thus, it may be an erroneous assumption to consider only stenoses of more than 60 or 70% luminal reduction clinically significant.

## Can we Reliably Assess the Functional Significance of Renal Artery Stenosis?

Most investigators would consider a renal artery stenosis hemodynamically significant only when the luminal diameter is reduced by at least 50 to 70%. Unfortunately, conventional imaging techniques such as CTA or MRA are less accurate in quantifying the degree of stenosis, although with adequate post-processing methods this may become better. Intravascular ultrasound (IVUS) and optical coherence tomography (OCT) are methods that allow direct visualization of the severity of the stenosis, but both require arterial catheterization and, as yet, cannot be applied on a large scale. Moreover, knowing to what extent the lumen is reduced still does not provide information on how severely the kidney may be affected by the given stenosis. Thus, one needs some functional test to assess the hemodynamic significance of a stenosis. Measurement of the intrarenal resistance index by Duplex ultrasonography has been proposed to serve that purpose, as there is a close correlation between the resistance index and the pressure gradient across the stenosis, at least in patients with a unilateral stenosis [23••]. However, even though Duplex ultrasonography is probably the best available non-invasive procedure at the moment, it has not gained widespread acceptance, largely because it requires specific operator skills. For years, on the other hand, clinicians have determined renal vein renin levels, but the renal vein renin ratio seems clinically useful only to detect patients with a totally occluded renal artery [24].

At the present time, more and more investigators tend to use measurements of the pressure gradients across the stenosis at baseline and during dopamine-induced hyperemia as an indicator of the hemodynamic significance of a stenosis [25, 26]. Still, there is no evidence that this is the best approach and, from a theoretical point of view, there are even arguments against putting too much trust in the results of such measurements. Indeed, what happens to a kidney with a stenotic artery depends not only on the degree of the stenosis, but also on how acutely this develops, and on the autoregulatory potential of the organ. When pressure distal to the stenosis is within the autoregulatory range and intrarenal vasodilation occurs to maintain renal blood flow, distal pressure will fall further thus creating, in fact, an impression of a tighter stenosis [27, 28]. In this case, the translesional pressure gradient will slightly increase. When the renin-angiotensin system is stimulated concurrently, the angiotensin II will raise intrarenal vascular resistance and, at least in part, offset such a rise in pressure gradient. Thus, measuring a pressure drop over a stenosis per se does not provide adequate information about the hemodynamic significance of the stenosis. Although it should be emphasized that the pathophysiology as outlined above has been derived from animal situations, there is no a priori reason to believe that these mechanisms are fundamentally different in humans. Often, the much-cited study by De Bruyne and co-workers is taken as evidence that renin production does not increase until the ratio of distal renal artery pressure to aortic pressure is lower than 0.9 [29]. However, this study is potentially flawed given that the investigators artificially created pressure gradients by balloon inflation in patients who already had a stenosis and had just received a stent. The results of that study, therefore, are applicable only to acute-on-chronic stenosis and not so much on stenosis per se. In a later study, the same group established that, although quantitative renal angiography and Doppler results correlate with the pressure ratio, both techniques tend to overestimate the degree of stenosis when the pressure ratio is taken as the gold standard to indicate a hemodynamically significant stenosis [30]. However, one could easily draw the opposite conclusion that a certain degree of anatomical stenosis may be associated with a lesser than expected pressure ratio, perhaps as a result of intrarenal vasoconstriction.

Recently, Van Brussel and associates reviewed the literature concerning basal and hyperemic hemodynamic measurements in patients with renal artery stenosis as a guide to treatment [31••]. In 11 of the 15 studies which they included in their analysis, the degree of renal arterial narrowing was reported to range from 51 to 78%. By and large, the correlation between anatomic severity of the stenosis, as determined angiographically, and hemodynamic data appeared to be relatively poor, and the predictive value of intrarenal functional data for outcome is still insufficient. Taken together, the available data strongly suggest that at the present, we do not yet have adequate tools to reliably establish the hemodynamic significance of any degree of ARAS in humans.

## Results of Revascularization Trials

Over the past 20 years, several randomized studies have been performed which evaluated the effect of percutaneous transluminal renal angioplasty without (PTRA) or with (PTRAS) stent placement over and above medical treatment compared to medical treatment alone. In a meta-analysis of these trials, angioplasty with or without stenting proved not to be superior to medical therapy alone with respect to a variety of outcome measures, including changes in systolic blood pressure, renal events, and cardiovascular complications [32••]. However, in 2016, an expert panel systematically reviewed all data from randomized as well as non-randomized studies concerning the comparative effectiveness and safety of PTRA plus stenting, surgical revascularization, and medical therapy to treat ARAS with regard to clinically important outcomes [33••]. The overall conclusion of this analysis was that the strength of the evidence that there is no or only a minimal clinically relevant difference between these treatments with regard to outcome or blood pressure control, is low. In other words, it remains uncertain whether mechanical treatment of the stenosis with or without stenting is beneficial or not.

Whenever a trial or a series of trials fail to show the expected results, and with the proviso that the interventional procedure(s) and the statistical analysis have been carried out appropriately, a likely conclusion may be that the pathophysiological concept upon which the trials are based is flawed and that selection of patients may have been suboptimal In all likelihood, this is the basic problem with the trials in ARAS that are available today. Indeed, the published trials all included patients with extensive disease, most of whom had high-grade stenosis and impaired renal function. This approach of selecting only patients with advanced abnormalities stems from the interpretation of pathophysiological data which show that lesser degrees of stenosis are not clinically or, for that matter, hemodynamically relevant. However, the threshold issue rests primarily on data obtained in experimental animals, which show that acute clipping of the renal artery has no measurable effect on renal blood flow or blood pressure at luminal reductions less than 70 to 80% [34]. In humans, on the other hand, renal artery stenosis develops much more slowly, allowing the kidney enough time to completely adapt to alterations in flow. Thus, setting a dividing line at some point to distinguish between significant and non-significant renal artery disease is arbitrary. With slowly progressive narrowing of its supplying artery, the kidney will mobilize compensatory (autoregulatory) mechanisms to safeguard as much function as possible. Only when the stenosis becomes too restrictive, these mechanisms become insufficient and renal function will decline. Therefore, patients who have reached that point, generally the type that has been included in the trials, must already have irreversible renal damage. Accordingly, it is no surprise that revascularization will not restore renal function in these cases [35]. We therefore conclude that the selection of patients for the angioplasty trials has not been optimal because these patients have little viable kidney tissue left and their chances to improve after revascularization were poor from the outset. In addition, and as pointed out repeatedly by others, we should move away from selecting patients purely on the basis of alleged hemodynamic alterations, but take into account also the various inflammatory and ischemia-related factors [14••, 15••].

## The Fallacy of the “Insignificant” Stenosis

The failure of the trials to show any significant clinical benefit should not lead to a nihilistic approach with respect to ARAS, but it should rather force us to adjust our ideas about whether our current thinking about the pathophysiology of renovascular disease is correct. Surely, the view that we should not be concerned about patients with low-grade stenosis is not tenable. As outlined above, there is enough data to show that even low-grade stenosis may be associated with a pressure gradient [36], and renal functional abnormalities already become apparent at a stenosis percentage around 30% [37]. Moreover, hypertensive patients with low-grade stenosis are at higher risk of cardiovascular complications as compared to hypertensive patients with patent renal arteries. Furthermore, they tend to have a faster decline in renal function than those with no abnormalities [13]. This tendency towards a faster decline in renal function in patients with a low-grade stenosis was also reported by others [38, 39]. It would appear, therefore, that there is no such thing as an insignificant renal artery stenosis.

## Conclusions

Patients with ARAS, be it low-grade or high-grade, have a prognosis that is worse than that of patients with patent renal arteries and even normotensive patients with this lesion are exposed to a greater risk of cardiovascular complications. Although there is a lack of studies specifically addressing the question whether patients with ARAS need more aggressive anti-atherosclerotic treatment than those without, it seems warranted that such a study will be carried out. In this regard, it is also relevant to delineate whether and at which degree of arterial narrowing mechanical treatment of the stenosis could confer most benefit. The major intervention trials have included only patients with high-grade stenosis (more than 50 or 60% luminal reduction), but these patients are already far underway on their path of atherosclerotic complications. There is ample evidence that even low-grade ARAS is already associated with pathophysiological changes in the kidney and the systemic vasculature. Naturally, these observations cannot be taken as proof that angioplasty in patients with low-grade stenosis is justified. From a scientific point of view, however, it is worthwhile to explore in a prospective trial whether in patients with low-grade stenosis angioplasty added to optimal anti-atherosclerotic treatment will produce a better outcome than medical treatment alone among patients with low-grade stenosis.

## References

[1] Kalra PA, Guo H, Kausz AT, Gilbertson DT, Liu J, Chen SC, Ishani A, Collins AJ, Foley RN (2005). Atherosclerotic renovascular disease in United States patients aged 67 years or older: risk factors, revascularization, and prognosis. Kidney Int.

[2] Uzu T, Takeji M, Yamada N, Fujii T, Yamauchi A, Takishita S (2002). Prevalence and outcome of renal artery stenosis in atherosclerotic patients with renal dysfunction. Hypertens Res.

[3] Conlon PJ, Athirakul K, Kovalik E, Schwab SJ, Crowley J, Stack R, McCants CB Jr, Mark DB, Bashore TM, Albers F (1998). Survival in renal vascular disease. J Am Soc Nephrol.

[4] Vassallo D, Green D, Ritchie J, Chrysochou C, Blunt J, Kalra PA (2016). Three decades of atherosclerotic reno-vascular disease management—changing outcomes in an observational study. Kidney Blood Press Res.

[5] Rimmer JM, Gennari FJ (1993). Atherosclerotic renovascular disease and progressive renal failure. Ann Intern Med.

[6] Wright JR, Shurrab AE, Cooper A, Kalra PR, Foley RN, Kalra PA (2005). Left ventricular morphology and function in patients with atherosclerotic renovascular disease. J Am Soc Nephrol.

[7] Zanoli L, Rastelli S, Marcantoni C, Capodanno D, Blanco J, Tamburino C, Laurent S, Boutouyrie P, Castellino P (2014). Non-hemodynamically significant renal artery stenosis predicts cardiovascular events in persons with ischemic heart disease. Am J Nephrol.

[8] Schwartz CJ, White TA (1964). Stenosis of renal artery: an unselected necropsy study. Br Med J.

[9] Holley KE, Hunt JC, Brown AL, Kincaid OW, Sheps SG (1964). Renal artery stenosis. A clinical-pathologic study in normotensive and hypertensive patients. Am J Med.

[10] Hansen KJ, Edwards MS, Craven TE, Cherr GS, Jackson SA, Appel RG, et al. Prevalence of renovascular disease in the elderly: a population-based study. J Vasc Surg. 2002;36(3):443–51. 10.1067/mva.2002.127351 12218965

[11] Mui KW, Sleeswijk M, van den Hout H, van Baal J, Navis G, Woittiez AJ (2006). Incidental renal artery stenosis is an independent predictor of mortality in patients with peripheral vascular disease. J Am Soc Nephrol.

[12] Fava C, Minuz P, Patrignani P, Morganti A (2006). Renal artery stenosis and accelerated atherosclerosis: which comes first?. J Hypertens.

[13] Dechering DG, Kruis HM, Adiyaman A, Thien T, Postma CT (2010). Clinical significance of low-grade renal artery stenosis. J Intern Med.

[14] Lerman LO, Textor SC (2015). Gained in translation: protective paradigms for the poststenotic kidney. Hypertension.

[15] Textor SC, Lerman LO (2015). Paradigm shifts in atherosclerotic renovascular disease: where are we now?. J Am Soc Nephrol.

[16] Eirin A, Gloviczki ML, Tang H, Rule AD, Woollard JR, Lerman A, et al. Chronic renovascular hypertension is associated with elevated levels of neutrophil gelatinase-associated lipocalin. Nephrol Dial Transplant. 2012;27(11):4153–61. 10.1093/ndt/gfs370. 10.1093/ndt/gfs370 PMC3616756 22923545

[17] Ronden RA (2013). Modulation of renal ADMA handling in hypertension. S’-Hertogenbosch: Maastricht University Medical Center.

[18] Schreiber MJ, Pohl MA, Novick AC (1984). The natural history of atherosclerotic and fibrous renal artery disease. Urol Clin North Am.

[19] Tollefson DF, Ernst CB (1991). Natural history of atherosclerotic renal artery stenosis associated with aortic disease. J Vasc Surg.

[20] Zierler RE, Bergelin RO, Isaacson JA, Strandness DE, Jr. Natural history of atherosclerotic renal artery stenosis: a prospective study with duplex ultrasonography. J Vasc Surg 1994;19(2):250–257; discussion 7-8. 10.1016/s0741-5214(94)70100-8 8114186

[21] Caps MT, Perissinotto C, Zierler RE, Polissar NL, Bergelin RO, Tullis MJ, et al. Prospective study of atherosclerotic disease progression in the renal artery. Circulation. 1998;98(25):2866–72. 10.1161/01.cir.98.25.2866 9860789

[22] Caps MT, Zierler RE, Polissar NL, Bergelin RO, Beach KW, Cantwell-Gab K, et al. Risk of atrophy in kidneys with atherosclerotic renal artery stenosis. Kidney Int. 1998;53(3):735–42. 10.1046/j.1523-1755.1998.00805.x 9507221

[23] Noory E, Rastan A, Beschorner U, Macharzina R, Zeller T (2016). Duplex derived intrarenal resistance index correlates with invasive pressure gradient measurements in detecting relevant unilateral renal artery stenosis. Vasa.

[24] Rossi GP, Cesari M, Chiesura-Corona M, Miotto D, Semplicini A, Pessina AC (2002). Renal vein renin measurements accurately identify renovascular hypertension caused by total occlusion of the renal artery. J Hypertens.

[25] Leesar MA, Varma J, Shapira A, Fahsah I, Raza ST, Elghoul Z, et al. Prediction of hypertension improvement after stenting of renal artery stenosis: comparative accuracy of translesional pressure gradients, intravascular ultrasound, and angiography. J Am Coll Cardiol. 2009;53(25):2363–71. 10.1016/j.jacc.2009.03.031. 10.1016/j.jacc.2009.03.031 19539148

[26] Mangiacapra F, Trana C, Sarno G, Davidavicius G, Protasiewicz M, Muller O, et al. Translesional pressure gradients to predict blood pressure response after renal artery stenting in patients with renovascular hypertension. Circ Cardiovasc Interv. 2010;3(6):537–42. 10.1161/CIRCINTERVENTIONS.110.957704. 10.1161/CIRCINTERVENTIONS.110.957704 21078879

[27] Anderson WP, Johnston CI, Korner PI (1979). Acute renal haemodynamic and renin-angiotensin system responses to graded renal artery stenosis in the dog. J Physiol.

[28] Anderson WP, Korner PI, Johnston CI (1979). Acute angiotensin II-mediated restoration of distal renal artery pressure in renal artery stenosis and its relationship to the development of sustained one-kidney hypertension in conscious dogs. Hypertension.

[29] De Bruyne B, Manoharan G, Pijls NH, Verhamme K, Madaric J, Bartunek J (2006). Assessment of renal artery stenosis severity by pressure gradient measurements. J Am Coll Cardiol.

[30] Drieghe B, Madaric J, Sarno G, Manoharan G, Bartunek J, Heyndrickx GR, et al. Assessment of renal artery stenosis: side-by-side comparison of angiography and duplex ultrasound with pressure gradient measurements. Eur Heart J. 2008;29(4):517–24. 10.1093/eurheartj/ehm631. 10.1093/eurheartj/ehm631 18276621

[31] van Brussel PM, van de Hoef TP, de Winter RJ, Vogt L, Van den Born BJ (2017). Hemodynamic measurements for the selection of patients with renal artery stenosis: a systematic review. JACC Cardiovasc Interv.

[32] •• Riaz IB, Husnain M, Riaz H, Asawaeer M, Bilal J, Pandit A, et al. Meta-analysis of revascularization versus medical therapy for atherosclerotic renal artery stenosis. Am J Cardiol. 2014;114(7):1116–23. 10.1016/j.amjcard.2014.06.033. **This paper summarizes the most recent trial data on the effectiveness of renal angioplasty and strenting**10.1016/j.amjcard.2014.06.033 25145333

[33] •• Balk EM, Raman G, Adam GP, Halladay CW, Langberg VN, Azodo IA et al. Renal artery stenosis management strategies: an updated comparative effectiveness review. Comparative effectiveness review no. 179. (prepared by the Brown evidence-based practice center under contract no. 290–2015-00002-I.), vol AHRQ publication no. 16-EHC026-EF. Rockville, MD: Agency for Healthcare and Quality; 2016. **Review by an expert panel on all available studies that investigated the effects of revascularization.**

[34] Haimovici H, Zinicola N (1962). Experimental renal-artery stenosis diagnostic significance of arterial hemodynamics. J Cardiovasc Surg.

[35] Koivuviita N, Liukko K, Kudomi N, Oikonen V, Tertti R, Manner I, et al. The effect of revascularization of renal artery stenosis on renal perfusion in patients with atherosclerotic renovascular disease. Nephrol Dial Transplant. 2012;27(10):3843–8. 10.1093/ndt/gfs301. 10.1093/ndt/gfs301 22785108

[36] Gross CM, Kramer J, Weingartner O, Uhlich F, Luft FC, Waigand J (2001). Determination of renal arterial stenosis severity: comparison of pressure gradient and vessel diameter. Radiology.

[37] Schreij G, Ritsema GH, Vreugdenhil G, de Leeuw PW (1996). Stenosis and renographic characteristics in renovascular disease. J Nucl Med.

[38] Cheung CM, Wright JR, Shurrab AE, Mamtora H, Foley RN, O’Donoghue DJ, et al. Epidemiology of renal dysfunction and patient outcome in atherosclerotic renal artery occlusion. J Am Soc Nephrol. 2002;13(1):149–57. 10.1681/ASN.V131149 11752032

[39] Myers DI, Poole LJ, Imam K, Scheel PJ, Eustace JA (2003). Renal artery stenosis by three-dimensional magnetic resonance angiography in type 2 diabetics with uncontrolled hypertension and chronic renal insufficiency: prevalence and effect on renal function. Am J Kidney Dis.

